# MF59- and Al(OH)_3_-Adjuvanted *Staphylococcus aureus* (4C-Staph) Vaccines Induce Sustained Protective Humoral and Cellular Immune Responses, with a Critical Role for Effector CD4 T Cells at Low Antibody Titers

**DOI:** 10.3389/fimmu.2015.00439

**Published:** 2015-09-07

**Authors:** Elisabetta Monaci, Francesca Mancini, Giuseppe Lofano, Marta Bacconi, Simona Tavarini, Chiara Sammicheli, Letizia Arcidiacono, Monica Giraldi, Bruno Galletti, Silvia Rossi Paccani, Antonina Torre, Maria Rita Fontana, Guido Grandi, Ennio de Gregorio, Giuliano Bensi, Emiliano Chiarot, Sandra Nuti, Fabio Bagnoli, Elisabetta Soldaini, Sylvie Bertholet

**Affiliations:** ^1^Research Center, Novartis Vaccines and Diagnostics S.r.l., Siena, Italy; ^2^Department of Biomedical Sciences, University of Padua, Padua, Italy; ^3^Department of Biology and Biotechnologies “Charles Darwin”, Sapienza University of Rome, Rome, Italy; ^4^Department of Biotechnology, Chemistry and Pharmacy, University of Siena, Siena, Italy

**Keywords:** *Staphylococcus aureus*, bacterial infection, vaccination, adjuvant, antibodies, T cells, mice

## Abstract

*Staphylococcus aureus* (*S. aureus)* is an important opportunistic pathogen that may cause invasive life-threatening infections, like sepsis and pneumonia. Due to the increasing antibiotic resistance, the development of an effective vaccine against *S. aureus* is needed. Although a correlate of protection against staphylococcal diseases is not yet established, several findings suggest that both antibodies and CD4 T cells might contribute to optimal immunity. In this study, we show that adjuvanting a multivalent vaccine (4C-Staph) with MF59, an oil-in-water emulsion licensed in human vaccines, further potentiated antigen-specific IgG titers and CD4 T-cell responses compared to alum and conferred protection in the peritonitis model of *S. aureus* infection. Moreover, we showed that MF59- and alum-adjuvanted 4C-Staph vaccines induced persistent antigen-specific humoral and T-cell responses, and protected mice from infection up to 4 months after immunization. Furthermore, 4C-Staph formulated with MF59 was used to investigate which immune compartment is involved in vaccine-induced protection. Using CD4 T cell-depleted mice or B cell-deficient mice, we demonstrated that both T and B-cell responses contributed to 4C-Staph vaccine-mediated protective immunity. However, the role of CD4 T cells seemed more evident in the presence of low-antibody responses. This study provides preclinical data further supporting the use of the adjuvanted 4C-Staph vaccines against *S. aureus* diseases, and provides critical insights on the correlates of protective immunity necessary to combat this pathogen.

## Introduction

*Staphylococcus aureus* (*S. aureus*) is an important opportunistic pathogen that causes skin and soft tissue infections as well as invasive life-threatening diseases, including sepsis and pneumonia. It is estimated that this Gram-positive bacterium causes 80,000 invasive infections each year in the US and about 15% of patients contracting invasive *S. aureus* succumb to this infection (1). Disease severity and increasing number of antibiotic-resistant strains urgently call for the development of an effective vaccine against this pathogen. So far, however, vaccines based on type 5 and 8 capsular polysaccharides or on a single conserved protein antigen (IsdB) have failed in Phase III and II/III clinical trials, respectively (2, 3).

Although a correlate of protection has not yet been established for staphylococcal infections, there are evidences that both humoral and cellular immunity are important to prevent staphylococcal diseases (4, 5). For example, immunocompromised individual with reduced ability to produce functional antibodies, such as acquired immune deficiency syndrome (6), or defects in immunoglobulin production (7), have increased susceptibility to staphylococcal infections. On the other hand, the help provided by CD4 T cells is required to develop functional antibody responses. Moreover, cytokines secreted by T helper cells like interferon-γ (IFN-γ) and IL-17 enhance recruitment and activation of neutrophils that are primary cellular defense against *S. aureus* infection and several groups have demonstrated that protection induced by vaccine candidates is mediated by these two cytokines in mouse animal models (1, 8, 9).

Based on the premises that humoral and cellular immune responses against multiple antigens might be needed to improve vaccine efficacy, we developed a protein-based vaccine (4C-Staph) targeting two surface-associated lipoproteins, FhuD2 and Csa1A, and three secreted virulence factors, α-Hemolysin (Hla), EsxA, and EsxB (10). FhuD2 is a lipoprotein involved in iron up-take and in early stages of invasive *S. aureus* infection (11–13). Csa1A is highly conserved across different *S. aureus* isolates and belongs to a family of proteins encoded in at least four distinct loci sharing from 54 to 91% sequence identity (14). FhuD2 and Csa1A were shown to confer protection in the abscess animal model (12, 14). Hla plays a role in the early stages of invasive and skin infections (15), and was shown to confer protection in several mouse infection models of *S. aureus*, including pneumonia (16, 17), peritonitis (10, 18), and dermonecrosis (15, 19). EsxA and EsxB are secreted through the ESAT-6 secretion system, which are involved in abscess formation (20–22). To be exploited as vaccine antigens, Hla was detoxified with a histidine to leucine substitution at position 35 (Hla_H35L_) (23), while EsxA and EsxB were fused together (EsxAB) (10). We previously demonstrated that 4C-Staph vaccine formulated with aluminum hydroxide (alum) was protective against several *S. aureus* strains in four animal models of infection: peritonitis, abscess formation, skin infection, and pneumonia (10, 24).

MF59 is an oil-in-water nano-emulsion licensed in 1997 to adjuvant human influenza vaccines and, to date, more than 150 million doses of MF59-adjuvanted vaccines have been administered worldwide. Preclinical and clinical studies showed that MF59 adjuvant enhances both the quantity and the quality of antigen-specific immune responses, allowing for antigen dose-sparing, improving the breadth of the antibody responses, and enhancing protective immunity in particular target populations, like children and the elderly (25).

Since the elderly is one of the populations most susceptible to *S. aureus* infection, we evaluated the adjuvant effect of MF59 on the immunogenicity and efficacy of 4C-staph vaccine *in vivo* and compared it to alum. Furthermore, we investigated the protective role of vaccine-specific antibody and CD4 T cells in B-cell deficient Jh mice or in mice depleted of CD4 T cells, respectively. Finally, the longevity of the protective immune responses induced by both vaccine formulations was evaluated by delaying the challenge up to 4 months after the last immunization.

## Materials and Methods

### Growth of *S. aureus* bacteria and mouse challenge studies

For mouse *in vivo* infection studies, *S. aureus* Newman strain (kindly provided by Professor Schneewind, University of Chicago) were grown in tryptic soy broth (Difco Laboratories) at 37°C with shaking until reaching mid-log phase. Bacteria were centrifuged, washed with PBS, and suspended in a volume of PBS to yield approximately 5 × 10^9^ colony-forming units (CFU)/ml. The inoculum was verified experimentally by plating on tryptic soy agar (Difco Laboratories) and colony enumeration the day after. Animals were challenged 2 weeks, 1 month or 4 months after the last immunization by intraperitoneal (i.p.) injection of *S. aureus* Newman (~5 × 10^8^ CFU). Mice were monitored for survival daily for 1 or 2 weeks. Survival curves were generated using GraphPad Prism 6.04 software (GraphPad Software). Protective efficacy was calculated as [1 − (% dead_4C-Staph/adjuvant_/% dead_adjuvant_)] × 100.

### Animals and immunization protocol

Female BALB/c or CD1 mice were purchased from Charles River Laboratories. Immunoglobulin (Ig) heavy chain-deleted Jh mice were purchased from Taconic. Five- to seven-week-old mice were used for all experiments. Mice were immunized intramuscularly (i.m.) with 10 μg of Hla_H35L_, FhuD2, EsxAB, and Csa1A (4C-Staph vaccine) in PBS, formulated 1:1 with MF59, or adsorbed to aluminum hydroxide adjuvant (alum, 2 mg/ml) in a total volume of 100 μl (50 μl/quadriceps). Control mice received an equal volume of saline, MF59 or alum adjuvant, respectively. Mice received one or two injections 2 weeks apart.

### Luminex assay

Animals were bled immediately prior to the immunization, and 2 weeks, 1 month, or 4 months after the last vaccination, and antigen-specific IgG present in sera of immunized mice were measured by Luminex technology (Luminex^®^ 200 TM). Hla_H35L_, FhuD2, EsxAB, and Csa1A-purified proteins were covalently conjugated to the free carboxyl groups of microspheres using a N-hydroxysulfosuccinimide-enhanced carbodiimide-mediated conjugation chemistry. Antigen-specific antibodies were revealed using phycoerythrin-labeled secondary antibodies. Data are expressed as fluorescence intensity (relative luminescence unit, RLU/ml) at a fixed serum dilution.

### Hla neutralization assay

Inhibition of Hla-induced hemolysis by functional anti-4C-Staph antibodies was evaluated in an *in vitro* assay as described elsewhere (10). Briefly, serial dilutions of sera from immunized mice were incubated with 12.5 nM Hla for 30 min at 37°C under constant agitation at 350 rpm. Erythrocytes derived from defibrinated rabbit blood were then added and the incubation prolonged for additional 30 min at 37°C. Incubation with water + 1% Triton X-100 was used as positive control (maximal hemolysis). Plates were then centrifuged for 5 min at 1,000 × *g* and the supernatant was analyzed by spectrophotometry at 540 nm using a SpectraMax^®^ 340PC384 Absorbance Microplate Reader (Molecular Devices).

### ELIspot analysis of Hla_H35L_-specific plasmablasts

Splenocytes from immunized mice were collected 2 weeks after the second vaccination. Multiscreen HTS_HA plates (96-well, Millipore) were coated with 100 μl of PBS containing 10 μg/ml of Hla_H35L_ or BSA, or 5 μg/ml of a goat anti-mouse Ig antibody (Southern Biotech), and blocked for 2 h at room temperature (RT) with PBS containing 10% fetal bovine serum. After three washing steps with PBS/Tween 20 0.05%, splenocytes (8 × 10^5^ cells/well) were seeded and incubated for 4 h at 37°C, 5% CO_2_. After six washing steps with PBS/Tween 20 0.05%, plates were incubated overnight with biotin-conjugated goat anti-mouse Ig (BD Pharmigen), followed by 30 min incubation with horseradish peroxidase-conjugated streptavidin (Endogen), and developed with the AEC substrate kit (Sigma) following the manufacturer’s protocol. Spots of antibody-secreting cells were counted using a UV Spot ELISpot plate Analyzer (CTL) and the Immunospot software v5.1 Professional DC (CTL).

### Intracellular flow cytometry

Splenocytes from immunized mice were isolated 2 weeks, 1 month or 4 months after the last immunization, plated at 1–2 × 10^6^ cells/well in 96-well plates, and stimulated with anti-CD28 and anti-CD49d (2 μg/ml each, BD Biosciences), EsxAB, Hla_H35L_, FhuD2 or Csa1A (30 μg/ml), or with a combination of antigens (4C-Staph) containing EsxAB, Hla_H35L_, FhuD2, and Csa1A (10 μg/ml each) at 37°C for 16–18 h, in the presence of Brefeldin A (5 μg/ml) for the last 4 h. The cells were then stained with Live/Dead Yellow (Invitrogen), fixed, and permeabilized with Cytofix/Cytoperm (BD Biosciences), washed in Perm/Wash buffer (BD Biosciences), incubated with anti-CD16/CD32 Fc block (BD Biosciences) for 20 min at RT, stained with fluorochrome-conjugated mAbs: anti-CD3-PerCP Cy5.5, anti-CD4-V500, anti-IFN-γ-PE, anti-IL-2-APC, anti-TNF-Alexa700, anti-CD44-V450 (BD Pharmingen), anti-CD8-PE Texas Red (Invitrogen), anti-IL-17 PE-Cy7, anti-IL-4-A488, and anti-IL-13-A488 (eBioscience), in Perm/Wash buffer 1× (BD Biosciences) for 20 min at RT, washed twice in Perm/Wash buffer, suspended in PBS. Samples were acquired on a LSRII special order flow cytometer (BD Biosciences) and T-cell responses were analyzed using FlowJo software (TreeStar) applying the gating strategy described in Figure S1 in Supplementary Material.

### T-cell proliferation

Splenocytes from immunized mice were collected at indicated time points, plated at 1 × 10^6^/well in 96-well plates, stimulated with EsxAB, Hla_H35L_, FhuD2, and Csa1A (10 μg/ml each) at 37°C 5% CO_2_ for 96 h and incubated for the last 24 h at 37°C 5% CO_2_ in the presence of 10 μM of Click-it^®^ Edu (Invitrogen). After two washing steps with PBS, cells were stained with Live/Dead Aqua (Invitrogen), incubated with anti-CD16/CD32 Fc block (BD Biosciences) for 20 min at RT in the dark and stained with anti-CD4 Pacific Blue and anti-CD44 APC (BD Biosciences). Cells were fixed with Cytofix (BD Biosciences), Click-it^®^ Edu detected following manufacturer’s instruction. Samples were acquired on a LSRII special order flow cytometer (BD Biosciences) and T-cell proliferation was analyzed using FlowJo software (TreeStar) applying the gating strategy described in Figure S1 in Supplementary Material.

### Depletion of CD4 T cells

To deplete CD4 T cells, mice were injected i.p. with 100 μg of rat anti-mouse CD4 antibody, clone GK1.5 (Areta International), or with the same amount of rat IgG2b isotype control antibody (R&D system) according to the schematics shown in Figure S2A in Supplementary Material.

- To abolish helper and effector CD4 T cells, animals received two injections of GK1.5 or isotype control antibodies before each immunization (on days 3 and 1, and on days 6 and 13, respectively).
- To abolish effector CD4 T cells, animals received two injections of GK1.5 or isotype control antibodies 4 and 2 days before infection. Ten days after immunization, mice were challenged with *S. aureus* as described above.

CD4 depletion efficiency was evaluated by flow cytometry on heparinized blood after red blood cell lysis with RBC lysis buffer (Biolegend). Cells were stained with Live/Dead Yellow (Invitrogen), washed in PBS, incubated with anti-CD16/CD32 Fc block (BD Biosciences) for 20 min at RT, stained with fluorochrome-conjugated mAbs: anti-CD3-PerCP Cy5.5 (BD Pharmingen), anti-CD8-PE Texas Red (Invitrogen) and anti-CD4 Pacific Blue (Invitrogen), in PBS 0.1% BSA for 20 min at RT, washed twice in PBS 0.1% BSA, fixed with Cytofix (BD Biosciences), washed twice and suspended in PBS. No CD4 T cells were detected in the peripheral blood indicating that depletion occurred efficiently (Figure S2B in Supplementary Material) and maintained overtime (Figure S2C in Supplementary Material).

### Statistical analyses

Statistical analyses were performed using GraphPad Prism 6.04 software (La Jolla, CA, USA). Experiments involving animal survival were analyzed by Mantle–Cox Log-rank test. Standard one-way ANOVA followed by Tukey’s or Dunn’s multiple comparison tests were used for analyses involving more than two treatment groups, unless otherwise indicated. The *p* values <0.05 were considered significant. Data reported in this work are representative of at least two experiments.

### Ethics statement

All animal studies were carried out in compliance with current Italian legislation on the care and use of animals in experimentation (Legislative Decree 116/92) and with the Novartis Animal Welfare Policy and Standards. Protocols were approved by the Italian Ministry of Health (authorization 249/2011-B). Following infection, mice were monitored daily and euthanized when they exhibited defined humane endpoints that were pre-established in agreement with Novartis Animal Welfare Policies.

## Results

### 4C-Staph antigens formulated with MF59 or alum confer protection against *S. aureus* infection in the peritonitis mouse challenge model

**Figure 1:**
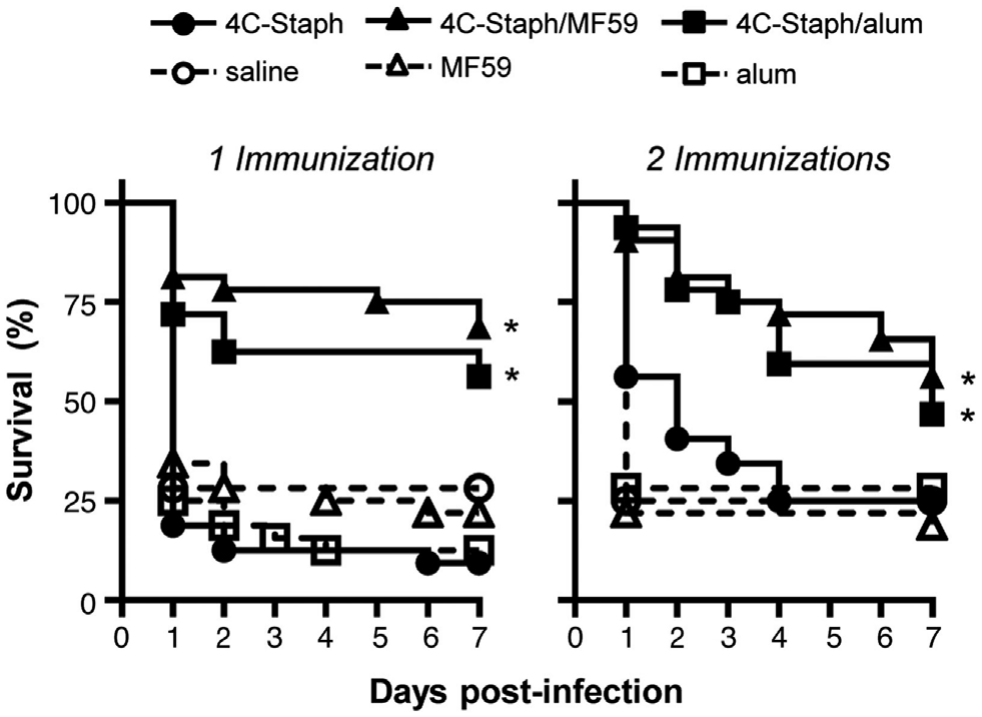
**4C-Staph formulations increased the survival of mice after *S. aureus* infection**. BALB/c mice were immunized with 4C-Staph, 4C-Staph/MF59, 4C-Staph/alum, and respective controls (PBS, MF59, and alum) once or twice 2 weeks apart, and challenged 2 weeks after the last immunization with ~5 × 10^8^ CFU *S. aureus*. Animals were monitored for 7 days post-infection for health status. Graphs represent the merge of at least two separate experiments (*n* = 32). Statistical analyses were performed using Mantel–Cox test; survival in the groups receiving the formulated 4C-Staph vaccines were compared to their respective control. *p < 0.05.

Efficacy of 4C-Staph vaccine was evaluated in the peritonitis model by immunizing mice once with 4C-Staph antigens alone, or in combination with MF59 or alum, or as control with saline or adjuvant alone. Two weeks after immunization, mice were infected i.p. with ~5 × 10^8^ CFU *S. aureus* and then monitored for up to 2 weeks. 4C-Staph/MF59- and 4C-Staph/alum-immunized mice showed significantly improved survival rates 7 days post-challenge compared to MF59 and alum adjuvant control, respectively (Figure 1 left panel). The increased survival induced by 4C-Staph/MF59 and 4C-Staph/alum was similar, while 4C-Staph alone showed lower efficacy with no statistical difference compared to control mice immunized with saline. As expected, neither MF59 nor alum adjuvant alone conferred protection against *S. aureus* challenge. Similarly, an increased survival was measured after two immunizations with 4C-Staph/MF59 and 4C-Staph/alum, while mice immunized with 4C-Staph alone did not show statistically significant differences with control mice immunized with saline (Figure 1 right panel). In the second week of observation, the survival decreased from 56 to 50% for 4C-Staph/MF59, from 46 to 40% for 4C-Staph/alum, and from 21 to 15% for 4C-Staph in the two immunizations regime (data not shown). In the single immunization regime, the survival decreased from 62 to 56% for 4C-Staph/MF59, from 56 to 53% for 4C-Staph/alum, and remained at about 10% for 4C-Staph. The protection for both adjuvanted vaccine formulations and schedules of immunization remained statistically significant compared to controls receiving adjuvant alone. These data suggest that one immunization is sufficient to induce a protective immune response against *S. aureus* infection, but only when the 4C-Staph antigens are adjuvanted with MF59 or alum. Since a longer time of observation did not add value to the results, survival data will be reported at 1 week post-infection for all the subsequent challenge experiments.

### Characterization of humoral responses induced by adjuvanted 4C-Staph antigens

Even though correlates of protection against *S. aureus* infection are yet to be established, there are evidences that antibodies are essential but not sufficient to prevent staphylococcal diseases. Passive transfer of hyper-immune sera directed against *S. aureus* antigens conferred protection *in vivo* in animal models, and *in vitro*, antibodies inhibited the function of virulence factors and toxins, for example, Hla (10).

**Figure 2:**
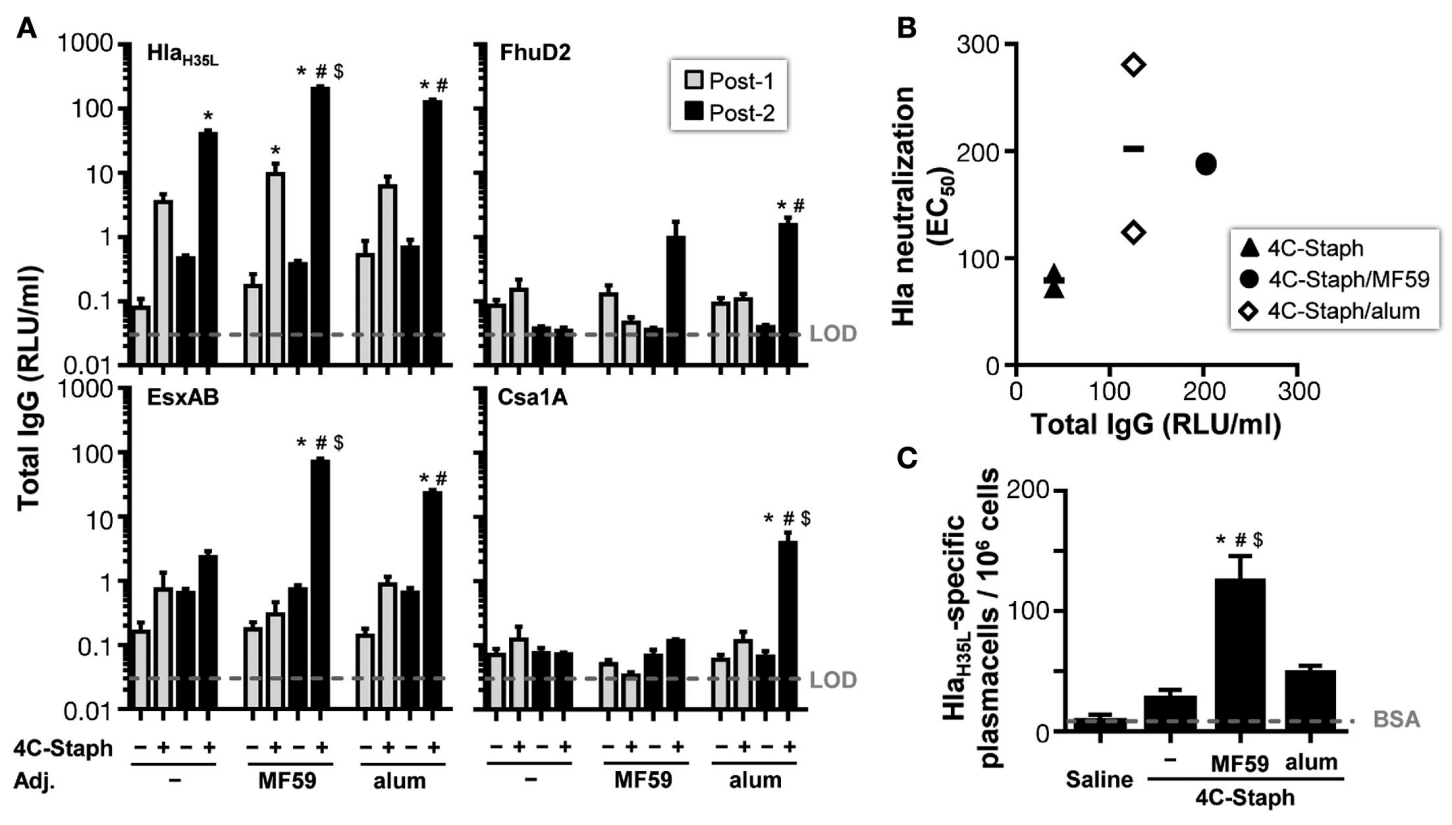
**4C-Staph formulations induced antigen-specific functional antibody and antibody-secreting B cells**. **(A)** IgG titers specific to each 4C-Staph component (Hla_H35L_, EsxAB, FhuD2, and Csa1A) were determined in sera of mice after one (Post-1) or two (Post-2) immunizations (*n* = 10–16). **(B)** Correlation between Hla-specific neutralization and antibody titers. **(C)** Hla_H35L_-specific antibody-secreting B cells were detected in spleen of mice 2 weeks after the second immunization (*n* = 4–8). Graphs show mean ± SD, and represent the merge of a least two separate experiments. Data were analyzed using one-way ANOVA and Tukey’s multiple comparison test, *p* < 0.05, *4C-Staph formulation versus its respective formulation without 4C-Staph; ^#^adjuvanted-4C-Staph versus 4C-Staph alone; ^$^4C-Staph/MF59 versus 4C-Staph/alum.

To characterize the immunogenicity of 4C-Staph formulations in term of antibody responses in mice, serum levels of IgG and IgM specific for each 4C-Staph antigen were measured after one or two vaccine administrations of 4C-Staph, 4C-Staph/MF59, and 4C-Staph/alum. Hla_H35L_-specific IgG titers were readily detectable after one immunization in all groups of vaccinated mice, and IgG titers in the 4C-Staph/MF59 group reached a significant difference versus control mice that received MF59 alone (Figure 2A). In contrast, low (EsxAB) to undetectable (FhuD2 and Csa1A) levels of antigen-specific IgG were observed in all 4C-Staph-vaccinated groups after a single immunization. The second dose boosted Hla_H35L_ and EsxAB-specific antibody titers in all groups, however, the difference in IgG titers after one and two immunizations reached statistical significance only for 4C-Staph/MF59 and 4C-Staph/alum. IgG isotypes were investigated for Hla_H35L_ 2 weeks after the second immunization. MF59- and alum-adjuvanted 4C-Staph vaccines elicited predominantly IgG1 (81 and 74%, respectively), followed by IgG2b (17 and 23%, respectively), and IgG2a (2 and 3%, respectively) (data not shown).

Hla_H35L_-specific IgM were detected in all vaccine groups at similar levels after a single immunization, and were not boosted by the second injection of 4C-Staph vaccine formulations (Figure S3 in Supplementary Material). In contrast, IgM responses against EsxAB, and IgM and IgG responses against FhuD2 and Csa1A were barely detectable, even after two immunizations.

Furthermore, the functional activity of Hla-specific antibodies induced by the different 4C-Staph vaccine formulations was tested in an *in vitro* neutralization assay and reported to the respective Hla-specific IgG titers. After two vaccine administrations, sera from all 4C-Staph-immunized mice showed detectable Hla-neutralizing titers (Figure 2B). Titers were similar in MF59- and alum-adjuvanted 4C-Staph formulations, and were slightly lower in the non-adjuvanted 4C-Staph group. Sera from mice vaccinated once had no detectable Hla-specific neutralizing activity (data not shown).

To correlate IgG and neutralization titers with B-cell frequencies, we evaluated the number of antigen-specific plasmablasts induced by the different 4C-Staph vaccine formulations. We observed that 4C-Staph/MF59 induced a higher frequency of Hla_H35L_-specific plasmablasts than 4C-Staph/alum and 4C-Staph alone (Figure 2C).

### Characterization of T-cell responses induced by adjuvanted 4C-Staph antigens

As several groups have demonstrated that T cells may play a role in vaccine-mediated protection (1, 8, 9), we characterized the functional profile of 4C-Staph-specific T cells induced by the different vaccine formulations by polychromatic flow cytometry and intracellular cytokine staining (ICS) assay. T cells were gated as CD3^+^CD4^+^CD44^hi^ cells after discrimination of singlets in live lymphocytes, defined morphologically by side scatter versus forward scatter plots (Figure S1A in Supplementary Material). The proliferation or production of IL-2, TNF, IFN-γ, IL-17, and IL-4/IL-13 was further determined on antigen-stimulated CD3^+^CD4^+^CD44^hi^ T cells (Figure S1B in Supplementary Material).

**Figure 3:**
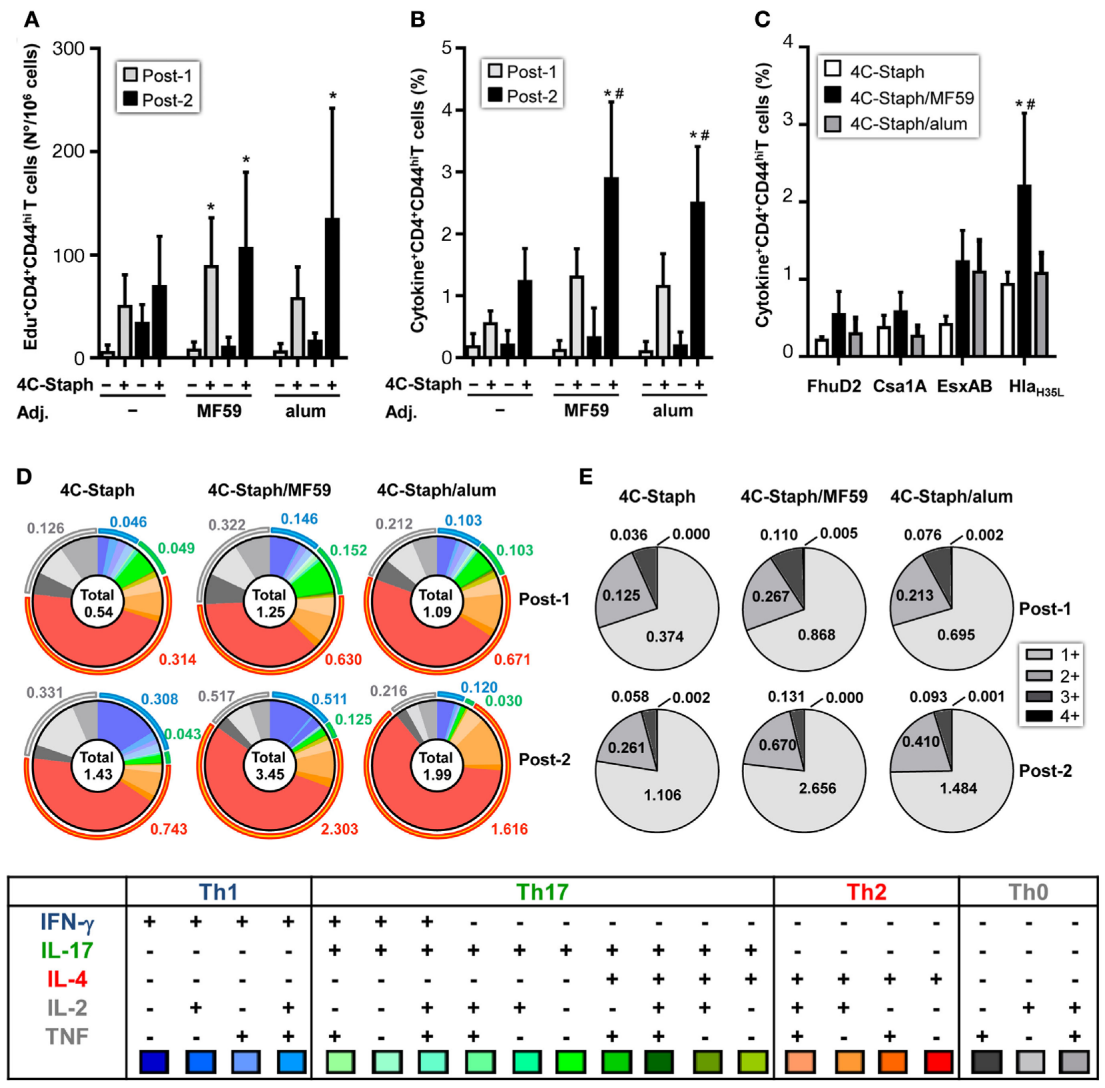
**4C-Staph formulations induced antigen-specific CD4 T cells**. Splenocytes from individual mice were stimulated *in vitro* with 4C-Staph antigens and Edu, IFN-γ, IL-4, IL-13, IL-17, IL-2, and/or TNF positive CD4^+^CD44^hi^ T cells were identified by flow cytometry using the gating strategy shown in Figure S1 in Supplementary Material. **(A)** Number of Edu^+^ proliferating CD4 T cells. **(B,C)** Frequency of cytokine^+^ CD4 T cells in response to stimulation with **(B)** all 4C-Staph antigens, or **(C)** with each single antigen (*n* = 10–18). **(D)** Pie charts representing polyfunctional CD4 CD44^hi^ T cells. **(E)** Frequencies of CD4 T cells expressing one or more cytokines. Graphs represent the merge of two separate experiments, *n* = 10. Graphs show mean ± SD. Data were analyzed using one-way ANOVA and Tukey’s multiple comparison test, *p* < 0.05, *4C-Staph formulation versus its respective formulation without 4C-Staph; ^#^adjuvanted-4C-Staph versus 4C-Staph alone; ^$^4C-Staph/MF59 versus 4C-Staph/alum.

4C-Staph-specific Edu^+^ proliferating CD4 T cells were observed after one immunization in 4C-Staph/MF59 and after two immunizations in 4C-Staph/MF59 and 4C-Staph/alum groups (Figure 3A). In addition, 4C-Staph-specific cytokine^+^ CD4 T cells were detected after one immunization in adjuvanted and non-adjuvanted 4C-Staph-immunized mice, even though to a lesser extent in mice that received non-adjuvanted 4C-Staph antigens and were boosted by the second immunization (Figure 3B). The highest frequency of total cytokine^+^ CD4 T cells was observed in 4C-Staph/MF59-immunized mice, 2 weeks after the second immunizations, followed by 4C-Staph/alum and 4C-Staph. Most of the cytokine^+^ CD4 T cells were reactive to Hla_H35L_ and EsxAB and to a lesser extent to FhuD2 and Csa1A (Figure 3C). When looking at the quality of the CD4 T-cell response, boolean gate analyses of the different cytokines (IL-2, TNF, IFN-γ, IL-17, and IL-4/IL-13) evidenced that, after one immunization, 4C-Staph/MF59 induced the highest number of IFN-γ^+^ (Th1) and IL-17^+^ (Th17) CD4 T cells, with frequencies of 0.146 and 0.152% of total CD4^+^CD44^high^ T cells, respectively, compared to 4C-Staph/alum (0.103% for both IFN-γ and IL-17) and 4C-Staph (0.046 and 0.049%, respectively) (Figure 3D). Frequencies of IL-2^+^, TNF^+^, and IL-2^+^TNF^+^ (Th0) CD4 T cells were also higher in the 4C-Staph/MF59 group compared to the other two groups, while 4C-Staph/alum induced the highest frequencies of IL-4/13^+^ (Th2) CD4 T cells, followed by 4C-Staph/MF59 and 4C-Staph. Two weeks after the second immunization, the magnitude of Th0, Th1, and Th2 CD4 T-cell responses increased in all three groups of vaccination, while Th17 decreased. 4C-Staph/MF59 induced the highest frequency of Th0, Th1, and Th17 combined (1.152%), followed by 4C-Staph (0.682%) and 4C-Staph/alum (0.339%). No significant differences in the proportion of CD4 T cells producing more than one cytokine were observed among 4C-Staph/MF59, 4C-Staph/alum, and 4C-Staph (Figure 3E).

Altogether, these data show that 4C-Staph vaccine-induced antigen-specific IgM, IgG, and pluri-functional CD4 T cells, and adjuvanting 4C-Staph antigens with MF59 or alum showed increased antibody titers and frequencies of CD4 T cells compared to non-adjuvanted antigens. 4C-Staph/MF59 vaccine formulation was selected to further characterize the immune mechanisms mediating protection against *S. aureus* infection.

### Contribution of 4C-Staph-specific antibodies and CD4 T cells to the protection against *S. aureus*

In order to determine the contribution of 4C-Staph-specific T cells and/or antibodies to the protection against *S. aureus* challenge, CD4 T cell-depleted BALB/c mice and Ig heavy chain deficient Jh mice were immunized once or twice with 4C-Staph/MF59 or with MF59 adjuvant alone as control. Two weeks after the last injection, mice were challenged with *S. aureus* Newman strain and their survival was monitored for 7 days.

**Figure 4:**
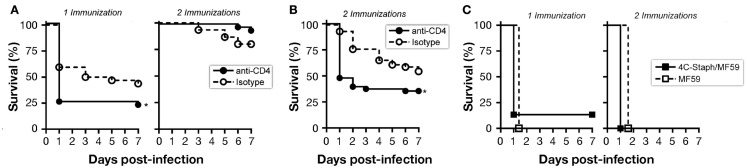
**B cells and CD4 T cells contribute to protection conferred by 4C-Staph formulations**. Mice were immunized once or twice with 4C-Staph/MF59 or MF59 and challenged with *S. aureus* 10 days after the last immunization. Animals were monitored for 7 days post-infection for health status. **(A,B)** CD4 T cells were depleted in immunized BALB/c mice by i.p. injection of anti-CD4 or isotype control antibodies (*n* = 48) **(A)** 1–3 days before challenge, or **(B)** 1 day before each immunization. **(C)** Survival of B-cell knock-out Jh mice (*n* = 15). Graphs represent the merge of three separate experiments. Statistical analyses were performed using Mantel–Cox test, *p < 0.05.

CD4 T cells may have a dual role: as (i) effector cells through the release of cytokines and/or (ii) providers of cognate help to B cells for the production of antigen-specific functional IgG. To evaluate a possible role for any of these two CD4 T-cell populations in 4C-Staph/MF59-mediated protection against *S. aureus*, we depleted CD4 T cells by treating mice with anti-CD4 antibody or an IgG2b isotype control before or after immunization (Figure S2A in Supplementary Material). Mice immunized once with 4C-Staph/MF59 and depleted of CD4 T cells after vaccination shortly prior to *S. aureus* challenge showed a significantly decreased survival compared to isotype control-treated mice, while no differences in survival were seen between anti-CD4 and isotype treatments in mice immunized twice (Figure 4A). These data suggest that effector CD4 T cells may have a prominent role in protecting mice against rapid death by *S. aureus* in the presence of low 4C-Staph-specific IgM and IgG titers (single immunization), while they might be dispensable at high antibody titers (two immunizations).

By depleting CD4 T cells before each of the two immunizations, 4C-Staph-specific CD4 T cells were not elicited, and IgG responses to all 4C-Staph antigens were decreased by up to two logs, while the frequency of CD4 T cells and magnitude of IgG responses in the isotype-treated group remained similar to that of untreated mice (Figures S2B and S4A in Supplementary Material). IgM titers were slightly reduced (Hla_H35L_ and EsxAB) or not affected (FhuD2 and Csa1A) in CD4 T cell-depleted mice compared to isotype-treated and untreated groups (Figure S4B in Supplementary Material). In these conditions, CD4 T cell-depleted mice were significantly more susceptible to *S. aureus* infection than isotype control-treated mice (Figure 4B), suggesting that 4C-Staph antigens are T cell-dependent antigens and that CD4 T cells are required for the development of the humoral response that in turn contributes to the control of *S. aureus* infection.

Finally, antibody-deficient Jh mice immunized once or twice with 4C-Staph/MF59 were not protected against a lethal challenge with *S. aureus*, dying within 48 h of infection, and showed the same survival rate than mice treated with MF59 alone (Figure 4C).

Altogether, these data suggest that the presence of 4C-Staph-specific antibody (IgG > IgM) is critical to the containment of *S. aureus* infection. Furthermore, in addition to their helper function, effector CD4 T cells may also participate to the mechanisms of protection when antibody titers are low.

### 4C-Staph vaccine induce persisting immunity and long-lasting protection

To evaluate the persistence of 4C-Staph-specific immunity, mice were vaccinated twice with 4C-Staph/MF59, 4C-Staph/alum, and the respective adjuvant alone as negative control. Sera and spleens were collected 1 month and 4 months after immunization to characterize 4C-Staph-specific antibody titers and CD4 T-cell responses, respectively.

**Figure 5:**
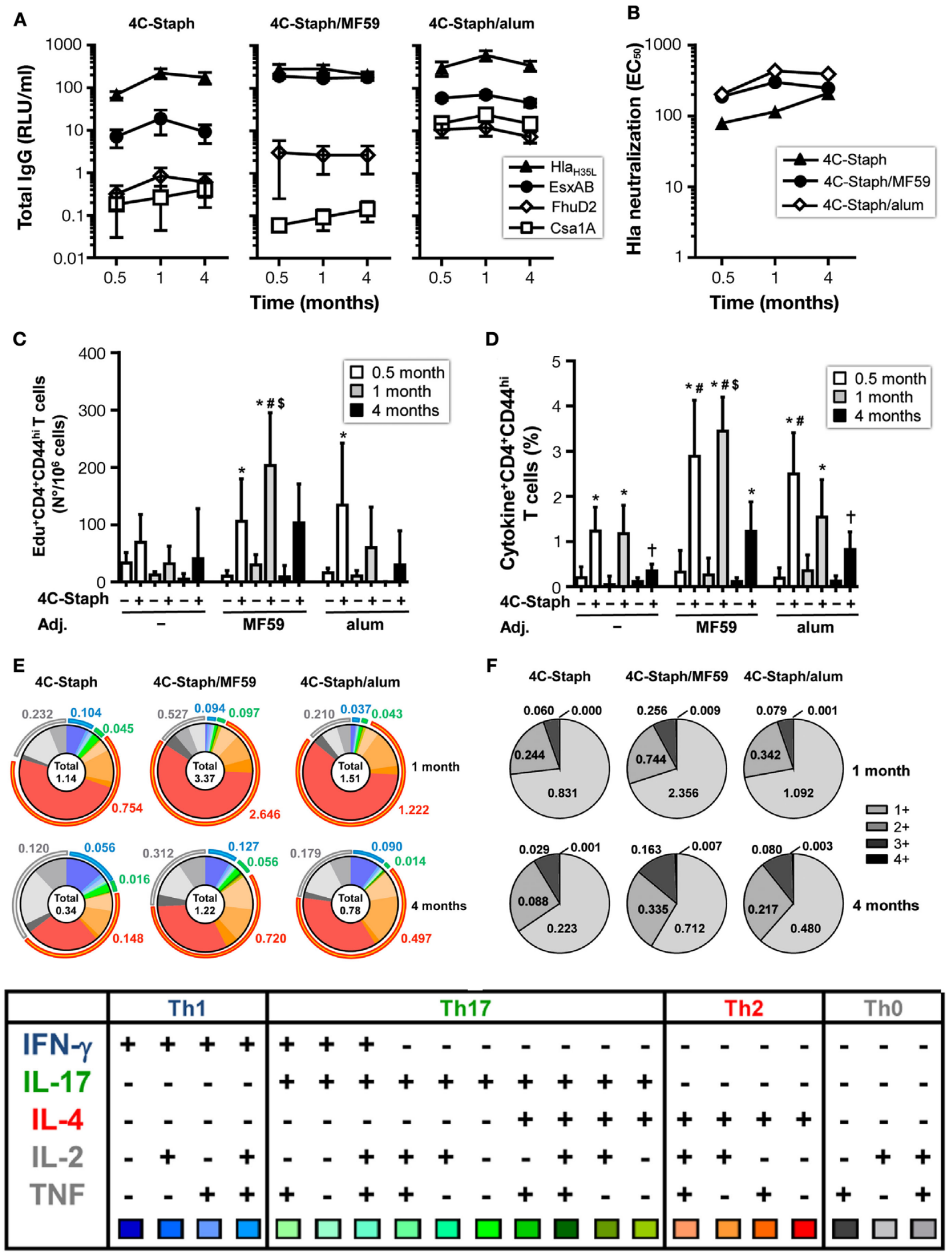
**Persistence of IgG and CD4 T-cell responses induced by 4C-Staph formulations**. BALB/c mice were immunized with 4C-Staph, 4C-Staph/MF59 and 4C-Staph/alum (*n* = 10), and immune responses were determined 0.5, 1, and 4 months after immunization. **(A)** IgG titers specific to each 4C-Staph component (Hla_H35L_, EsxAB, FhuD2, and Csa1A) (*n* = 10–16). **(B)** Hla-specific neutralization titers. Number of **(C)** Edu^+^ proliferating and frequency of **(D)** cytokine^+^ CD4 T cells in response to *in vitro* stimulation with 4C-Staph antigens. **(E)** Pie charts representing polyfunctional CD4 CD44^hi^ T cells. **(F)** Frequencies of CD4 T cells expressing one or more cytokines. Graphs show mean ± SD, and are the merge of at least two separate experiments. Data were analyzed using one-way ANOVA and Tukey’s multiple comparison test, *p* < 0.05, *4C-Staph formulation versus its respective formulation without 4C-Staph; ^#^adjuvanted-4C-Staph versus 4C-Staph alone; ^$^4C-Staph/MF59 versus 4C-Staph/alum; ^†^1 month or 4 months versus 0.5 month time point.

4C-Staph-specific antibody titers were sustained in 4C-Staph-, 4C-Staph/MF59-, and 4C-Staph/alum-immunized mice up to 4 months after vaccination (Figure 5A). Titers remained higher in the two 4C-Staph-adjuvanted formulations at all time points evaluated. The magnitude of IgG responses of each vaccine formulation was antigen-dependent: alum formulation was more immunogenic than MF59 for Csa1A and FhuD2, MF59 was more immunogenic for EsxAB, while alum- and MF59-adjuvanted vaccines were equally immunogenic against Hla. Hla-neutralizing antibody titers were detected in all vaccinated groups 1 month after the second vaccine administration, and were sustained up to 4 months with no significant differences among non-adjuvanted and adjuvanted-4C-Staph formulations (Figure 5B).

One month after vaccination, frequencies of 4C-Staph-specific Edu^+^ and cytokine^+^ CD4 T cells were further increased in the 4C-Staph/MF59 group. In contrast, the number of proliferating CD4 T cells were reduced in 4C-Staph and 4C-Staph/alum groups, while frequencies of cytokine^+^ CD4 T cells were maintained in the 4C-Staph group and were reduced in the 4C-Staph/alum group (Figures 5C,D). At 4 months, the magnitude of CD4 T-cell responses decreased in all groups receiving the 4C-Staph antigens, reaching frequencies similar or lower to those seen 2 weeks after two immunizations for 4C-Staph/MF59 and 4C-Staph/alum, respectively. Analysis of the cytokines secreted by 4C-Staph-specific CD4 T cells mainly showed a contraction of IL-4^+^ CD4 T cells between 1 and 4 months (Figure 5E). Furthermore, the frequencies of IL-17-producing T cells decreased 4 months after the second immunization (Figure 5E) compared to 2 weeks after the first injection (Figure 3D). Overall, the proportion of CD4 T cells producing 2 and 3 cytokines increased from 1 to 4 months (Figure 5F).

**Figure 6:**
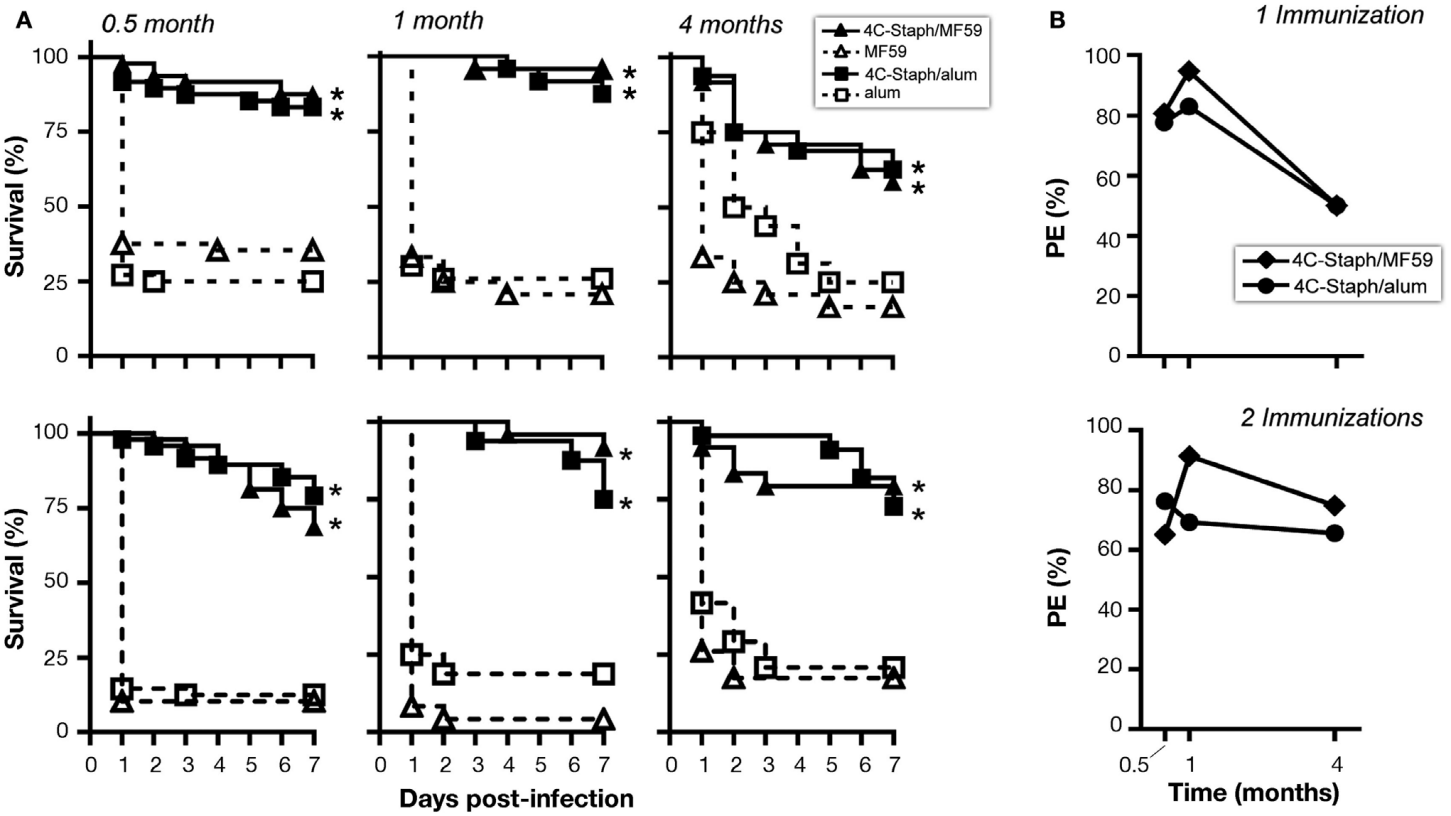
**4C-Staph formulations induced persistent protection against *S. aureus***. CD1 mice were immunized once or twice with 4C-Staph, 4C-Staph/MF59, and 4C-Staph/alum (*n* = 10) and challenged with *S. aureus* 0.5, 1, or 4 months after immunization. Animals were monitored for 7 days post-infection for health status. **(A)** Survival and **(B)** protective efficacy (PE) calculated (as described in Materials and Methods) on day 7 post-infection. Graphs represent the merge of at least two separate experiments (*n* = 16–24). Statistical analysis was performed using Mantel–Cox test; survival in the groups receiving the formulated 4C-Staph vaccines were compared to their respective control. *p < 0.05.

Finally, to evaluate whether the immune responses observed at later time point from vaccination were still protective, mice immunized once or twice with 4C-Staph/MF59 or 4C-Staph/alum were challenged 1 month or 4 months after vaccination with *S. aureus* and their survival was monitored for 7 days. 4C-Staph/MF59- and 4C-Staph/alum-immunized mice were still protected when challenged 1 or 4 months post-vaccination (Figure 6A). Protective efficacy (calculated as described in Materials and Methods) was higher at 1 month than 2 weeks after vaccination and sustained at least up to 4 months with both 4C-Staph/MF59 and 4C-Staph/alum formulations (Figure 6B). Moreover, 4C-Staph/MF59 outperformed 4C-Staph/alum at late time points. Similar results were observed if mice were infected after one or two vaccine injections. Altogether, these data suggest that 4C-Staph vaccine formulations induced levels of protective immune responses sustained at least for 4 months after immunization.

## Discussion

*Staphylococcus aureu*s is one of the most frequent causes of bacterial infection in humans. Despite the pressing medical need and the continued efforts, the development of a *S. aureus* vaccine remains a difficult goal not yet achieved. In the present study, we formulated 4C-Staph with MF59, an adjuvant licensed in human influenza vaccines targeting the elderly, a population also at higher risk of *S. aureus* infections. We demonstrated that both MF59- and alum-formulated 4C-Staph vaccines elicited persisting protective immune responses, and increased the survival rates after a lethal challenge with S*. aureus* in a mouse peritonitis model of infection. 4C-Staph/MF59 induced high levels of antigen-specific IgM, IgG, plasmablasts, CD4 T cells (Th0/Th1/Th17) and long-term protection. Dissecting the mechanism(s) of 4C-Staph/MF59-mediated protection, using B-cell deficient and CD4 T cell-depleted mice, revealed that 4C-Staph-specific antibodies were critical to the containment of *S. aureus* infection, while the contribution of effector CD4 T cells was most pronounced at low antibody titers. Altogether, these data suggest that, in the *S. aureus* peritonitis model, a vaccine formulation inducing both antibodies and T cells might be preferred.

The inability of adjuvanted and non-adjuvanted 4C-Staph vaccine formulations to protect Jh mice from *S. aureus* lethal infection confirmed the importance of antibodies in protecting against *S. aureus* infection as already reported in animal models (10, 12) and also supported by human studies (26–29). However, several vaccines against *S. aureus* infection that elicited high antibody titers (3, 30) failed in phase III clinical trials, despite encouraging preclinical results (3, 30–32). In addition, evidences supporting a role for T cell-mediated immunity in host defense against *S. aureus* infections are emerging in human studies and in animal models. For example, patients with poorly controlled HIV and low CD4 T-cell counts have high rates of *S. aureus* skin and soft tissue infections [reviewed in Ref. (33)]. Furthermore, patients with the Hyper-IgE syndrome, in which Th17 function is impaired, are highly susceptible to *S. aureus* skin and lung infections (34), as are mice that are deficient in IL-17 (35, 36). Th17 cells have also proven to be relevant in the protection conferred by IsdB vaccine against lethal *S. aureus* i.v. infection (9). Recently, Montgomery et al. showed that both antibody and Th17 responses were conferring protective immunity in a mouse model of skin infection (37). Therefore, targeting T-cell responses against *S. aureus*, in addition to humoral responses, may be critical in developing protection against infection. In this study, we report that a single immunization of non-adjuvanted 4C-Staph antigens elicited CD4 T-cell responses whose magnitude increased in the presence of an adjuvant, and were further boosted by the second vaccine administration. By means of multiparameter flow cytometry, we characterized the quality of the T-cell responses following immunization and showed that the highest number of antigen-specific Th0, Th1, and Th17 cells was induced by 4C-Staph/MF59. In an effort to establish the contribution of antigen-specific CD4 T cells to the protection conferred by 4C-Staph/MF59, CD4 T cells were depleted in immunized mice. CD4 T-cell depletion experiments demonstrated that CD4 T cells were crucial to the development of antigen-specific humoral responses and in turn to vaccine-induced protection. In addition to their helper function, effector CD4 T cells also contributed to protection against *S. aureus*, probably through the production of cytokines that enhanced the recruitment and activation of innate immune cells, such as neutrophils, key players in the control of *S. aureus* infection (38). The role of effector CD4 T cells was more evident when antibody titers were low (CD4 T-cell depletion after a single immunization), while their contribution to protection was not appreciable in presence of high antibody titers (CD4 T-cell depletion after two immunizations). Notably, the frequency of IL-17-producing CD4 T cells was the highest after one immunization. These observations may suggest a role for IL-17^+^ CD4 T cells in protection against *S. aureus*, in agreement with published data (9, 34–36, 39, 40). Surprisingly, we showed that the percent survival was not different in mice that received one or two vaccine administrations, despite significantly different antibody titers. On the other hand, we detected a Th1/Th17 bias more pronounce after one immunization that could be in turn responsible for vaccine-induced protection after a single vaccination. We can speculate that in conditions of sub-optimal humoral responses, the role of CD4 T cells, and in particular Th1/Th17 responses, become more evident in contributing to protection against bacterial infection. Along these lines, we recently reported that 4C-Staph formulated with alum and a TLR7 agonist small molecule immunopotentiator (4C-Staph/T7-alum) shifted CD4 T cell polarization toward Th1/Th17 and significantly increased protection against *S. aureus* infection compared to 4C-Staph/alum (10). While T7-alum adjuvant might hold great promise in the future, it is not readily available as safety in humans has yet to be confirmed.

Taken together, the results presented in this study demonstrated that rationally designed first generation 4C-Staph vaccine, formulated with alum or MF59, two adjuvants licensed in human vaccines, induced persistent protection, albeit partial, in an animal model of *S. aureus*-induced peritonitis, and that 4C-Staph-specific antibodies and CD4 T cells contributed to the protective mechanisms.

## Author Contributions

EM, FM, GL, MB, ST, CS, LA, MG, BG, SRP, and AT performed experiments. EM, MF, GB, EC, ES, FB, EG, GG, SN, and SB designed the study and analyzed the data. EM and SB wrote the manuscript.

## Conflict of Interest Statement

All authors were employees of or affiliated with Novartis Vaccines and Diagnostics S.r.l. at the time the study was performed.

## Supplementary Material

The Supplementary Material for this article can be found online at http://journal.frontiersin.org/article/10.3389/fimmu.2015.00439

## Abbreviations

alum, aluminum hydroxide; CFU, colony-forming units; LOD, limit of detection; RLU, relative luminescence units; *S. aureus*, *Staphylococcus aureus*.
