# Supplementary material for: MF59- and Al(OH)3-Adjuvanted Staphylococcus aureus (4C-Staph) Vaccines Induce Sustained Protective Humoral and Cellular Immune Responses, with a Critical Role for Effector CD4 T Cells at Low Antibody Titers
Source: Front Immunol. 2015 Sep 7;6:439. doi: 10.3389/fimmu.2015.00439 (PMC4561515; doi:10.3389/fimmu.2015.00439)
Supplement: Supplementary file 1 [file Presentation_1.PDF]

FIGURE S1

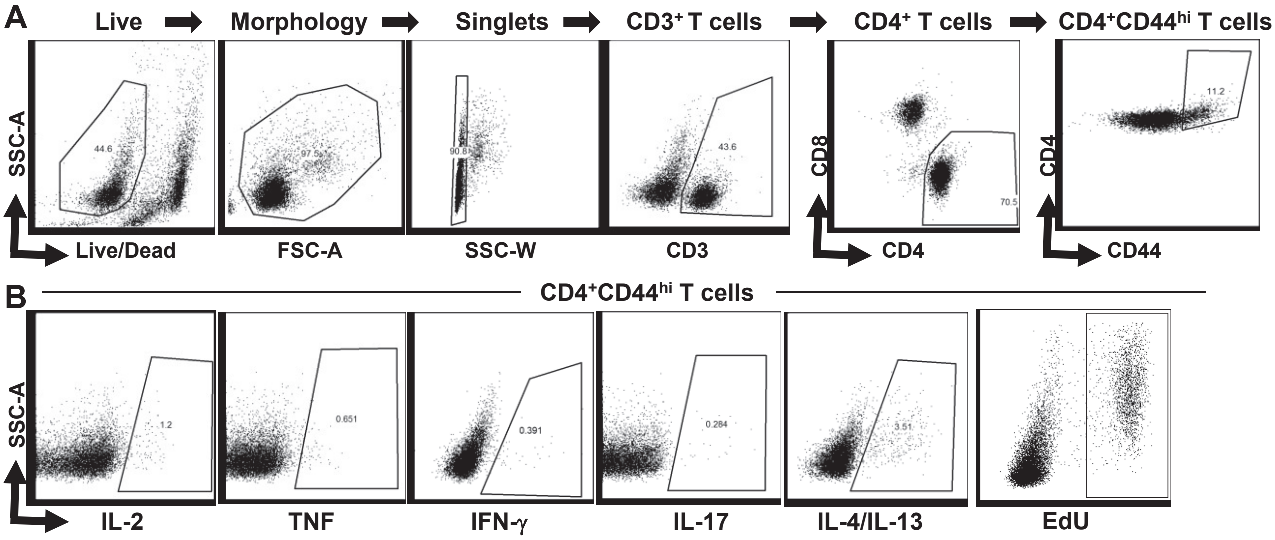

FIGURE S1. Gating strategy for flow cytometry analyses of CD4 T cells. (A) Live cells were negatively selected based on dye exclusion, and lymphocytes were further identified based on morphology. $CD3^{+}$ T cells were selected after discrimination of singlets and CD4 T cells were identified based on $CD4^{+}$ and $CD8^{-}$ expression. (B) Proliferating (Edu^{+}) and cytokine (IFN-γ, IL-4, IL-13, IL-17, IL-2 and TNF) positive cells were identified among $CD4^{+}CD44^{hi}$ T cells. Data shown are representative dot plots.

FIGURE S2

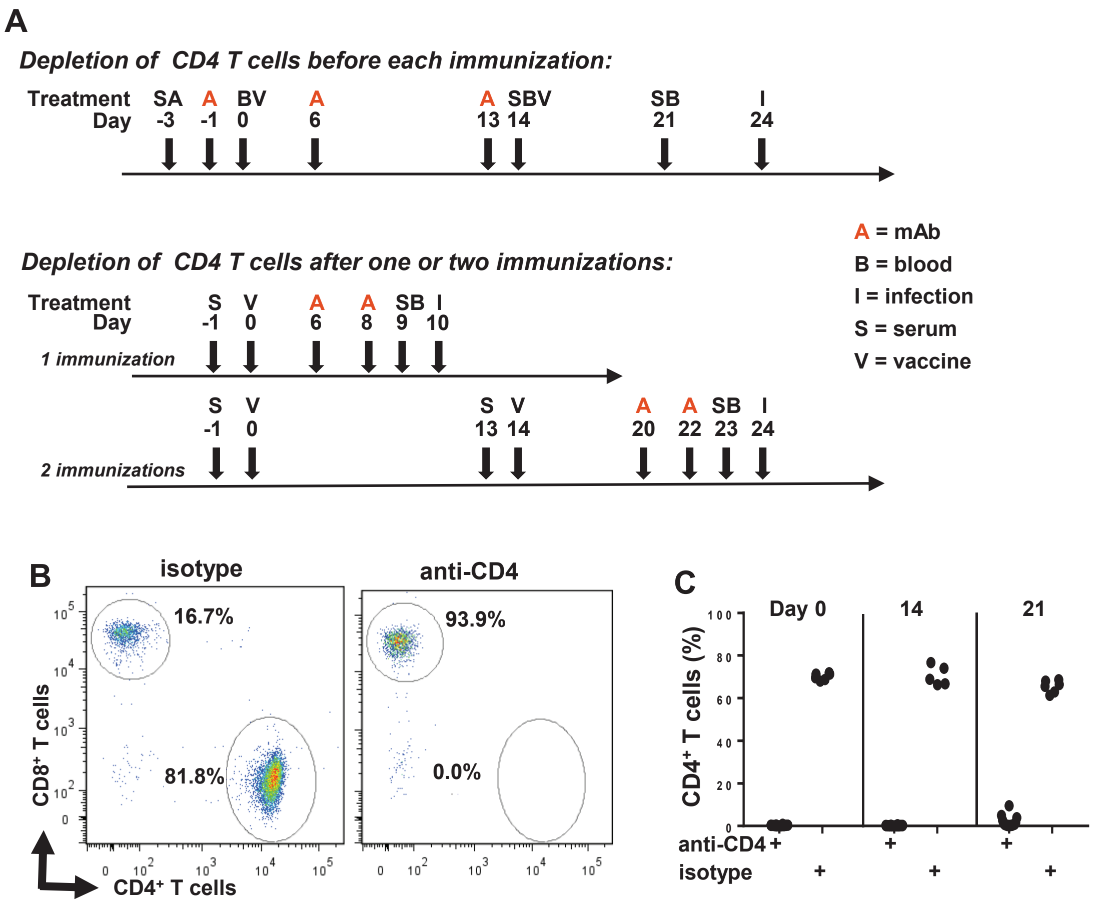

FIGURE S2. Depletion CD4 T cells. (**A**) Schematics of treatments. (**B**) Depletion efficacy in peripheral blood of mice treated with anti-CD4 or isotype antibody demonstrated by flow cytometry analyses. Blood was collected from individual mice at the indicated time points and CD4 T cells were identified on live cells based on the expression of CD3, CD4 and CD8 markers. Data shown are representative dot plots the day after the second antibodies injection. (**C**) Frequency of CD4 T cells in peripheral blood of mice after treatment with anti-CD4 or isotype control antibody over time. Data shown are single mice in a representative experiment.

FIGURE S3

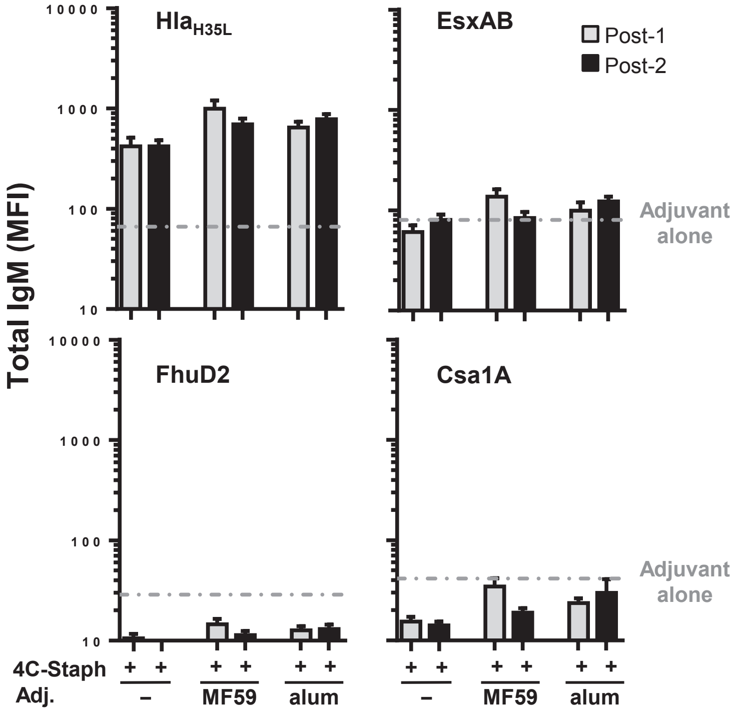

FIGURE S3. 4C-Staph formulations induced antigen-specific IgM. IgM titers specific to each 4C-Staph component (Hla_{H35L}, EsxAB, FhuD2 and Csa1A) were determined in sera of mice after one (Post-1) or two (Post-2) immunizations (n=10-16). Graphs show mean ± SEM and represent the merge of a least two separate experiments. Data were analyzed using one-way ANOVA and Tukey's multiple comparison test, $p < 0.05$, *4C-Staph formulation versus its respective formulation without 4C-Staph; $^{\#}$adjuvanted-4C-Staph versus 4C-Staph alone; $^{\$}$4C-Staph/MF59 versus 4C-Staph/alum.

FIGURE S4

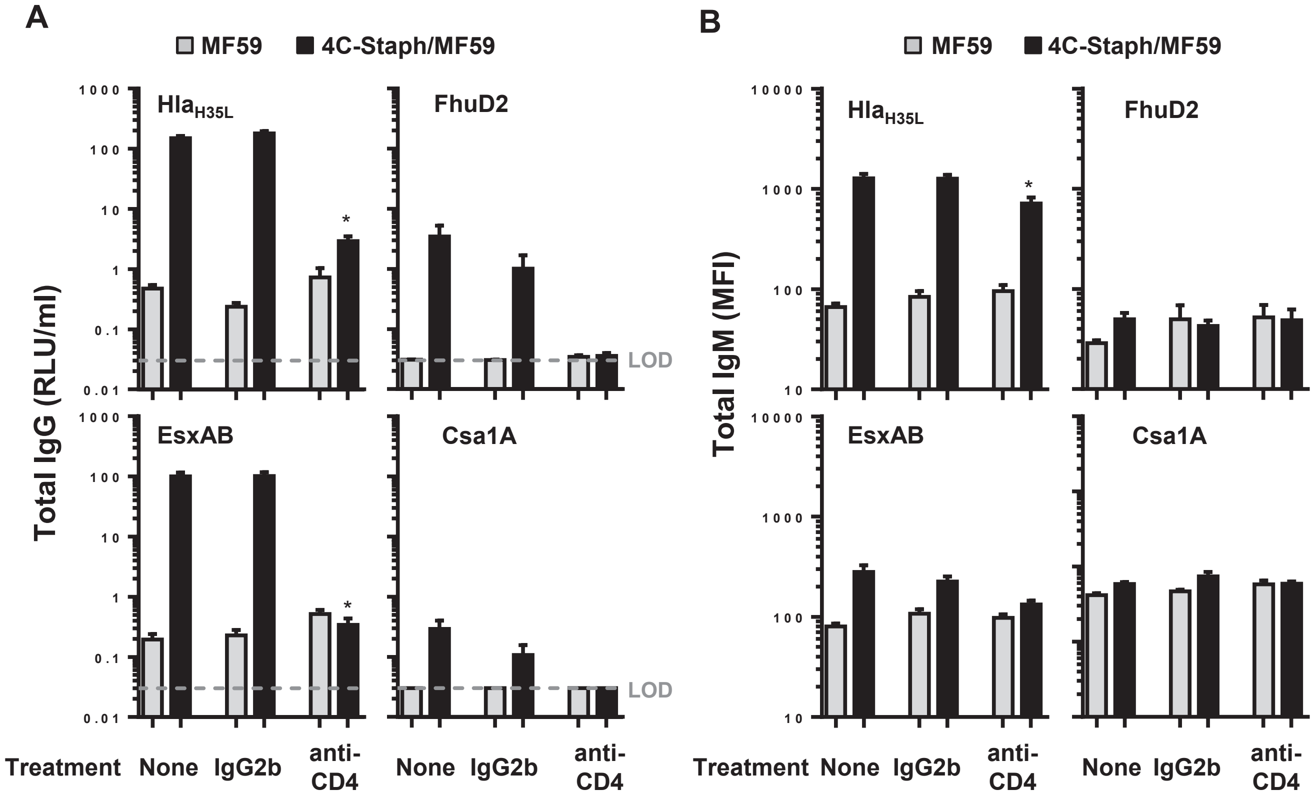

FIGURE S4. Effect of CD4 T-cell depletion before immunization on 4C-Staph - specific IgG and IgM titers. Sera from 4C-Staph/MF59- and MF59-immunized mice were collected on day 21 after treatment with anti-CD4 or isotype control antibody and two immunizations. (**A**) IgG and (**B**) IgM antibody titers against each 4C-Staph component (Hla_{H35L}, EsxAB, FhuD2 and Csa1A) were determined. Data show mean ± SEM and represent the merge of two separate experiments, (n=32). Data were analyzed using one-way ANOVA and Tukey's multiple comparison test. *p<0.05, anti-CD4 versus isotype control treatment.
